# Impact of *FOSL1* polymorphisms and expression on disease progression in oral tongue squamous cell carcinoma

**DOI:** 10.7150/jca.142594

**Published:** 2026-10-16

**Authors:** Ping-Ju Chen, Yi-Tzu Chen, Chun-Yi Chuang, Chun-Wen Su, Shun-Fa Yang, Ying-Erh Chou, Chiao-Wen Lin

**Affiliations:** 1 Department of Dentistry, Changhua Christian Hospital, Changhua, Taiwan.; 2 Department of Post-Baccalaureate Medicine, College of Medicine, National Chung Hsing University, Taichung, Taiwan.; 3 Institute of Medicine, Chung Shan Medical University, Taichung, Taiwan.; 4 School of Dentistry, Chung Shan Medical University, Taichung, Taiwan.; 5 Department of Dentistry, Chung Shan Medical University Hospital, Taichung, Taiwan.; 6 Institute of Oral Sciences, Chung Shan Medical University, Taichung, Taiwan.; 7 School of Medicine, Chung Shan Medical University, Taichung, Taiwan.; 8 Department of Otolaryngology, Chung Shan Medical University Hospital, Taichung, Taiwan.; 9 Department of Medical Research, Chung Shan Medical University Hospital, Taichung, Taiwan.

**Keywords:** oral tongue squamous cell carcinoma, FOSL1, polymorphism, betel quid

## Abstract

Oral tongue squamous cell carcinoma (OTSCC) is the most common subtype of oral cancer, with betel quid chewing, tobacco smoking, and alcohol consumption recognized as major risk factors. This study investigated the associations between FOSL1 single-nucleotide polymorphisms (SNPs) and disease susceptibility and clinicopathological characteristics in OTSCC patients. Three FOSL1 SNPs (rs637571, rs653914, and rs10896065) were genotyped in 377 male OTSCC patients and 1,194 controls using real-time polymerase chain reaction. The *FOSL1* rs10896065 polymorphic T variant was significantly associated with advanced clinical stage (Stage III-IV; AOR = 1.663, 95% CI = 1.059-2.611, p = 0.027) and larger tumor size (AOR = 1.813, 95% CI = 1.162-2.831, p = 0.009). Among betel quid chewers, carriers of the CT+TT genotype showed significant associations with advanced clinical stage, larger tumor size, and moderate-to-poor tumor differentiation. Similar associations with advanced stage and greater tumor size were observed among smokers carrying the CT+TT genotype. Analysis of The Cancer Genome Atlas dataset further demonstrated that elevated FOSL1 mRNA expression was significantly associated with larger tumor size (T3-T4 vs. T1-T2, p = 0.0216). These findings suggest that the *FOSL1* rs10896065 T allele is associated with advanced clinicopathological features in OTSCC patients, particularly among betel-quid chewers and smokers. *FOSL1* polymorphisms may have potential as biomarkers associated with clinicopathological characteristics in OTSCC.

## Introduction

Oral squamous cell carcinoma (OSCC) is the most prevalent head and neck malignancy, and the oral tongue squamous cell carcinoma (OTSCC) is the most frequent subtype 1, 2. In Taiwan, most oral cancer patients are male, and the development of oral cancer is strongly influenced by lifestyle and environmental risk factors, such as alcohol consumption, betel quid chewing, and cigarette smoking 3, 4.

FOS-like antigen 1 (FOSL1), also known as FOS-related antigen 1 (FRA1), is a member and a transcription factor of the activator protein 1 (AP1) complex and is localized to the cell cytoplasm and nucleus 5-7. Epigenetic activation of AP1 was suggested to promote squamous cell carcinoma metastasis 8. FOSL1 is not only a key component of the AP1 transcriptional complex but has also been implicated in many biological processes including cell migration, invasion, proliferation, cell plasticity, and cell cycle 5. Recent studies have suggested that FOSL1 is markedly upregulated and correlated with malignant progression in head and neck squamous cell carcinoma (HNSCC) 8-11.

A previous study has suggested that a functional single-nucleotide polymorphism (SNP) rs1892901 in *FOSL1* might affect the expression of FOSL1, and ultimately increase the risk of gastric cancer 12. In desmoplastic fibroblastoma, *FOSL1* was suggested as a candidate target gene for 11q12 rearrangements 13. However, the associations of *FOSL1* polymorphisms with OTSCC progression and clinicopathologic characteristics remain unclear. In this study, we examined three SNPs of *FOSL1* rs637571, rs653914, and rs10896065, and investigated their associations with OTSCC susceptibility and clinicopathologic characteristics in relation to environmental risk factors, including betel quid chewing and smoking.

## Materials and Methods

### Study subjects

A total of 377 male patients with OTSCC and 1,194 cancer-free controls were enrolled in this study. The OTSCC patients were clinically staged at the time of diagnosis according to the 8th edition of the American Joint Committee on Cancer (AJCC) Cancer Staging Manual. Demographic information, including age and sex, was obtained from each participant. Age was categorized as <60 years and ≥60 years for the statistical analyses. Information regarding betel-quid chewing, cigarette smoking, and alcohol drinking was obtained from participant interviews and/or medical records. Betel-quid chewing and cigarette smoking were classified according to exposure status, including ever and never exposure, and information regarding duration and quantity of exposure was collected where available. The control group consisted of individuals without self-reported diseases. This study was approved by the Institutional Review Board of Chung Shan Medical University Hospital (CSMUH No. CS1-21151), and written informed consent was obtained from all participants.

### Sample preparation and DNA extraction

In this study, we collected the peripheral blood specimens from normal controls and OTSCC patients for genomic DNA extraction 14. Each sample of peripheral whole blood was preserved with EDTA containing tubes. The samples of peripheral whole blood were centrifuged at 3,000 rpm for 10 minutes. The centrifuged buffy coats from the whole blood specimens were collected and further used for DNA extraction. For the DNA isolation, the genomic DNA extraction assay was performed according to the manufacturer's instructions of QIAamp DNA blood mini kits. The final DNA elution was completed with Tris-EDTA (TE) buffer. The extracted DNA was used as a DNA template in the following real-time polymerase chain reactions (PCRs).

### Selection of *FOSL1* SNPs and* FOSL1* SNPs genotyping

In our study, we selected three *FOSL1* SNPs rs637571, rs653914, and rs10896065 based on the International HapMap Project database 15. Based on the dbSNP database, the minor allele frequencies (MAFs) of rs637571 (C>T), rs653914 (A>G), and rs10896065 (C>T) in the Asian population were 0.250 (T), 0.489 (G), and 0.150 (T), respectively. Assessment of allelic discrimination for the *FOSL1* rs637571 (assay IDs: C___2867106_10), rs653914 (assay IDs: C___2867095_10), and rs10896065 (assay IDs: C___2867098_10) SNP was performed with an ABI StepOne Software v2.3 Real-Time PCR System. The TaqMan assay was applied for the analysis of genotyping. Genotyping data were analyzed using SDS 7000 series software (Applied Biosystems, Foster City, CA, USA).

### TCGA data analysis

Gene expression and clinical data for patients with OSCC were obtained from The Cancer Genome Atlas (TCGA) database 16. The analysis was restricted to patients with tumors arising from the oral tongue and base of tongue. FOSL1 mRNA expression was analyzed using RSEM-normalized expression values, and expression levels were presented as mean ± standard deviation (SD). For overall survival analysis, patients were dichotomized into high- and low-FOSL1-expression groups using the median FOSL1 expression value as the cutoff, resulting in 74 patients in each group (n = 148). Kaplan-Meier analysis was used to compare overall survival between the high- and low-expression groups, and differences were evaluated using the log-rank test.

### Statistical analysis

To compare demographic, lifestyle, and clinicopathological characteristics between OTSCC patients and controls, Student's t-test was used for continuous variables, and the chi-squared test was used for categorical variables. Continuous variables are presented as mean ± standard deviation, and categorical variables are presented as numbers and percentages. Hardy-Weinberg equilibrium with biallelic marker was tested using the Chi-Square goodness-of-fit test. To evaluate the associations between *FOSL1* genotypes and the clinicopathological characteristics of OTSCC, multivariable logistic regression models were used to estimate odds ratios (ORs) and corresponding 95% confidence intervals (CIs). The regression models were adjusted for potential confounding variables, including age, alcohol consumption, smoking, and betel-quid chewing duration, as appropriate for the respective analyses. Age was treated as a categorical variable and categorized as <60 years and ≥60 years. The reference group for each categorical variable was specified in the corresponding tables. All statistical analyses were performed using SAS statistical software, Version 9.1 (SAS Institute Inc., Cary, NC, USA).

## Results

The distribution of demographical characteristics in 1194 controls and 377 male patients with OTSCC is listed in Table 1. In the current study, we observed that the number of patients aged < 60 years was 776 (65.0%) in controls and 241 (63.9%) in OTSCC patients, and the age ≥ 60 in controls and OTSCC patients were 418 (35.0%) and 136 (36.1%), respectively. The prevalence of environmental exposure among controls and patients with OTSCC were 198 (16.6%) and 249 (66.0%) in betel quid chewing (p < 0.001), 635 (53.2%) and 289 (76.7%) in cigarette smoking (p < 0.001), and 237 (19.8%) and 141 (37.4%) in alcohol drinking (p < 0.001), respectively.

The genotype distributions of *FOSL1* gene polymorphisms in 1194 controls and 377 OTSCC patients are listed in Table 2. The most frequently observed genotypes for the *FOSL1* polymorphisms rs637571, rs653914, and rs10896065 in male patients with OTSCC were CC, AG, and CC, respectively. Genotypic distributions of these three *FOSL1* SNPs in the control group conformed to Hardy-Weinberg equilibrium (χ2 value=1.514, p=0.219 for rs637571; χ2 value=0.126, p=0.722 for rs653914, and χ2 value=0.208, p=0.648 for rs10896065). The adjusted odds ratios (AOR) with their 95% confidence intervals (CIs) were estimated by multiple logistic regression models. After adjustment for the effects of age, betel quid chewing, cigarette smoking, and alcohol drinking, no significant associations were identified between the *FOSL1* polymorphisms rs637571, rs653914, and rs10896065 and OTSCC susceptibility in the study population (Table 2).

We further analyzed the associations between clinical characteristics and the genotypic frequencies of the *FOSL1* rs637571, rs653914, and rs10896065 SNPs in 377 OTSCC patients. Although no significant associations were observed between the *FOSL1* rs637571 and rs653914 polymorphisms and clinicopathological characteristics in OTSCC patients (Table 3), OTSCC patients carrying the *FOSL1* rs10896065 variant genotype (CT + TT) were significantly more likely to present with advanced clinical stage (Stage III + IV) [AOR (95% CI): 1.663 (1.059-2.611); p = 0.027] and larger tumor size [AOR (95% CI): 1.813 (1.162-2.831); p = 0.009] (Table 3).

Moreover, among the 377 OTSCC patients stratified by betel quid chewing status, significant associations were observed in betel quid chewers carrying the *FOSL1* rs10896065 variant genotype (CT + TT). These individuals exhibited significant associations with advanced clinical stage (Stage III + IV) [AOR (95% CI): 2.030 (1.133-3.637); p = 0.017], larger tumor size [AOR (95% CI): 2.107 (1.187-3.740); p = 0.011], and moderate-to-poor tumor differentiation [AOR (95% CI): 3.549 (1.032-12.201); p = 0.044] (Table 4). Similarly, among the 377 OTSCC patients stratified by smoking status, smokers carrying the *FOSL1* rs10896065 variant genotype (CT + TT) showed significant associations with advanced clinical stage (Stage III + IV) [AOR (95% CI): 1.800 (1.066-3.042); p = 0.028] and larger tumor size [AOR (95% CI): 1.751 (1.048-2.926); p = 0.033] (Table 5).

To further investigate the clinical significance of FOSL1 in OSCC, data from the TCGA cohort of oral tongue and base-of-tongue squamous cell carcinoma patients were analyzed (Figure 1A-1D). FOSL1 mRNA expression was significantly higher in patients with larger primary tumors (T3 + T4) than in those with smaller tumors (T1 + T2) (p = 0.0216; Figure 1B). In contrast, no significant differences in FOSL1 mRNA expression were observed between patients with early-stage (Stage I + II) and advanced-stage (Stage III + IV) disease (Figure 1A, p = 0.2549) or between patients with and without lymph node metastasis (Figure 1C, p = 0.5545). Moreover, Kaplan-Meier survival analysis showed a non-significant trend in overall survival according to FOSL1 expression level, although the difference between patients with high and low FOSL1 expression did not reach statistical significance (p = 0.096; Figure 1D).

## Discussion

In this study, we revealed the associations between the *FOSL1* SNPs and male patients with OTSCC. More than 90% of malignant tumors diagnosed in the oral cavity are OSCC, and the tongue is the most common location 2. Etiological factors such as tobacco smoking, smokeless tobacco use, alcohol consumption, and betel quid chewing are well-established for OTSCC, and these risk factors were suggested to be synergistically increasing the carcinogenic risk 17. Consistent with these results, in our current study, statistically significant associations of these risk factors including betel quid chewing, cigarette smoking, and alcohol drinking were found in 377 male patients with OTSCC compared with 1194 controls, respectively.

Oral submucous fibrosis (OSF) is a precancerous lesion of the oral cavity, and areca (betel) nut consumption can cause OSF through the sustained activation of buccal mucosal fibroblasts (BMFs) 18, 19. For the correlations of betel quid chewing and FOSL1, a previous study has suggested that curcumin targets FOSL1 to reduce MAPK8 expression, thereby suppressing arecoline-induced fibroblast activation 18. Besides, curcumin was found to suppress HNSCC tumorigenesis through directly targeting FOSL1/JUN 20, suggesting the vital role of FOSL1 overexpression in OSF and HNSCC tumorigenesis. These findings further support the importance of aberrant molecular regulation in the pathogenesis of OSCC and provide a broader context for investigating transcriptional regulators such as FOSL1 in oral carcinogenesis.

Compared with these findings, although the precise role of the *FOSL1* rs10896065 in cancer progression and disease prognosis remains unclear, it is possible that, in OTSCC patients who are betel quid chewers or smokers, this polymorphic variant may be associated with FOSL1 overexpression through transcriptional activation, potentially mediated by a super-enhancer (SE)-driven transcriptional program. Further functional studies are warranted to elucidate the biological mechanisms underlying these gene-environment interactions in OTSCC progression.

Previous studies have suggested that the oncogenic function of FOSL1 was linked to KRAS and ectopic activation of ERK 21. The constitutive activation of ERK and FOSL1 could promote various aspects of tumor progression, including enhancing cancer cell survival, facilitating tumor proliferation, and promoting epithelial-mesenchymal transition (EMT) 21-24. In hepatocellular carcinoma (HCC), FOSL1 mRNA expression in HCC was significantly higher than that in adjacent non-tumor tissues, suggesting a potential role of FOSL1 in HCC tumorigenesis 22. In triple-negative breast cancer (TNBC), FOSL1 was identified as a pivotal glycolysis-related transcription factor which promotes tumor growth in TNBC in a glycolysis-dependent manner 25. Compared with these findings, our analysis of the TCGA dataset demonstrated that higher FOSL1 mRNA expression was significantly associated with larger tumor size in OSCC, but not with clinical stage or lymph node metastasis. This discrepancy between the genetic association of *FOSL1* rs10896065 and the TCGA expression profiles may reflect differences between genetic variation and gene expression, as well as differences in patient characteristics and tumor heterogeneity. Further studies are needed to clarify the role of rs10896065 in OTSCC progression. In addition, *FOSL1* rs10896065 variant carriers showed significant associations with advanced clinical stage and larger tumor size among individuals exposed to environmental risk factors, such as betel quid chewing or smoking. However, these associations were not statistically significant among non-chewers or non-smokers. These findings suggest that the association between the *FOSL1* rs10896065 polymorphism and advanced clinicopathological features in OTSCC may be more evident in individuals exposed to betel quid chewing or smoking. However, the underlying biological mechanism remains unclear.

Several limitations of this study should be acknowledged. First, we did not directly evaluate whether rs10896065 affects FOSL1 expression or transcriptional activity. FOSL1 mRNA expression data were not available in OTSCC patients stratified by rs10896065 genotype, particularly among carriers of the CT + TT genotypes. Thus, a direct association between rs10896065 and FOSL1 expression could not be established. The proposed mechanisms, including enhancer-mediated transcriptional regulation, environmental interactions, and FOSL1 overexpression, should therefore be considered hypothetical. No eQTL analysis or functional validation, such as reporter assays, was performed. Further genotype-stratified expression analyses and functional studies are needed to determine whether rs10896065 directly regulates FOSL1 expression. Second, this was a single-center study conducted in Taiwan and restricted to male patients, which may limit the generalizability of the findings. The hospital-based recruitment design may also have introduced selection bias, and residual confounding cannot be completely excluded. In addition, the limited sample sizes in some genotype and subgroup categories may have reduced statistical power for stratified analyses. Finally, the findings have not been independently replicated in an external cohort. Larger, multi-centre studies including both sexes, as well as independent replication and functional validation, are warranted.

In conclusion, this study demonstrated the associations between *FOSL1* polymorphisms and clinicopathological characteristics in OTSCC patients. The *FOSL1* rs10896065 variant genotype was significantly associated with advanced clinical stage and larger tumor size among both betel quid chewers and smokers. Collectively, these findings suggest that *FOSL1* polymorphisms are associated with advanced clinicopathological features in OTSCC. Further studies are warranted to determine the underlying biological mechanisms and their potential relevance to disease progression.

## Figures and Tables

**Figure 1:**
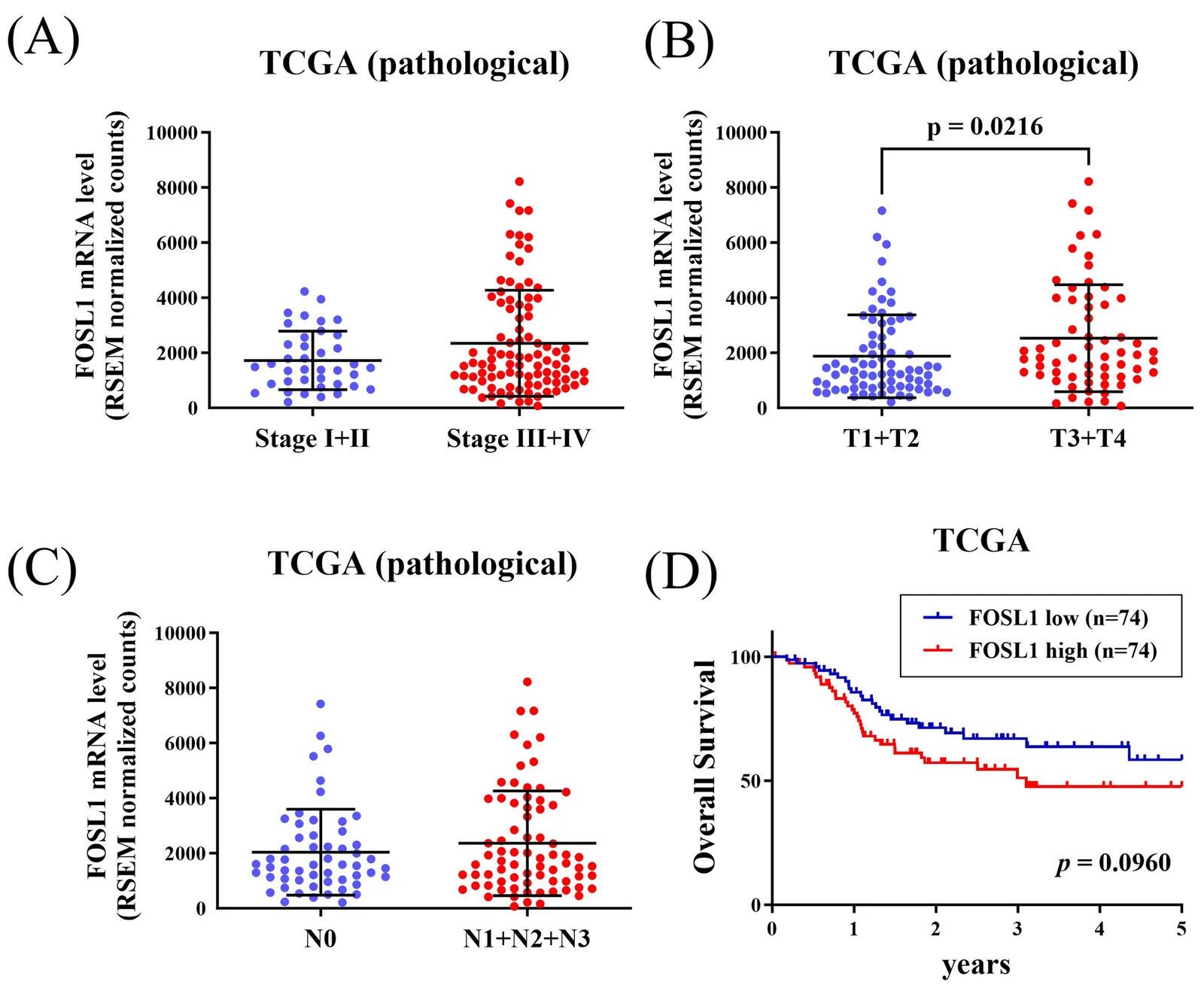
** Association between FOSL1 expression and clinicopathological characteristics and survival outcomes in TCGA OSCC patients (oral tongue and base of tongue).** (A) Comparison of FOSL1 mRNA expression between patients with early-stage (Stage I + II) and advanced-stage (Stage III + IV) disease (p = 0.2549). (B) Comparison of FOSL1 mRNA expression between patients with smaller (T1 + T2) and larger (T3 + T4) primary tumors, with significantly higher expression observed in T3 + T4 tumors (p = 0.0216). (C) FOSL1 mRNA expression according to lymph node status (N0 vs. N1 + N2 + N3) (p = 0.5545). FOSL1 mRNA expression is presented as RNA-Seq by Expectation-Maximization (RSEM)-normalized expression values, with data shown as mean ± standard deviation (SD). (D) Kaplan-Meier survival analysis of overall survival in patients with high and low FOSL1 expression, showing a non-significant trend (p = 0.096).

**Table 1:** The distributions of demographical characteristics in 1194 controls and 377 male patients with oral tongue squamous cell carcinoma (OTSCC).

Variable	Controls (N=1194)	Patients (N=377)	p value
**Age (yrs)**			
< 60	776 (65.0%)	241 (63.9%)	p=0.706
≥ 60	418 (35.0%)	136 (36.1%)	
**Betel quid chewing**			
No	996 (83.4%)	128 (34.0%)	
Yes	198 (16.6%)	249 (66.0%)	p <0.001*
**Cigarette smoking**			
No	559 (46.8%)	88 (23.3%)	
Yes	635 (53.2%)	289 (76.7%)	p <0.001*
**Alcohol drinking**			
No	957 (80.2%)	236 (62.6%)	
Yes	237 (19.8%)	141 (37.4%)	p <0.001*
**Stage**			
I+II		167 (44.3%)	
III+IV		210 (55.7%)	
**Tumor T status**			
T1+T2		193 (51.2%)	
T3+T4		184 (48.8%)	
**Lymph node status**			
N0		230 (61.0%)	
N1+N2+N3		147 (39.0%)	
**Distant metastasis**			
M0		375 (99.5%)	
M1		2 (0.5%)	
**Cell differentiation**			
Well differentiated		42 (11.1%)	
Moderately or poorly differentiated		335 (88.9%)	

* p value < 0.05 as statistically significant. Note: All participants in both the OTSCC patient and control groups were male.

**Table 2:** Odds ratio (OR) and 95% confidence interval (CI) of oral tongue squamous cell carcinoma associated with *FOSL1* genotypic frequencies.

Variable	Controls (N=1194) (%)	Patients (N=377) (%)	AOR (95% CI)	p value
**rs637571**				
CC	721 (60.4%)	214 (56.8%)	1.000 (reference)	
CT	423 (35.4%)	140 (37.1%)	1.147 (0.869-1.513)	p=0.333
TT	50 (4.2%)	23 (6.1%)	1.469 (0.810-2.666)	p=0.205
CT+TT	473 (39.6%)	163 (43.2%)	1.183 (0.906-1.543)	p=0.217
**rs653914**				
AA	322 (27.0%)	104 (27.6%)	1.000 (reference)	
AG	602 (50.4%)	192 (50.9%)	0.885 (0.648-1.208)	p=0.442
GG	270 (22.6%)	81 (21.5%)	0.905 (0.621-1.319)	p=0.603
AG+GG	872 (73.0%)	273 (72.4%)	0.901(0.671-1.209)	p=0.486
**rs10896065**				
CC	843 (70.6%)	257 (68.2%)	1.000 (reference)	
CT	318 (26.6%)	110 (29.2%)	1.309 (0.975-1.758)	p=0.073
TT	33 (2.8%)	10 (2.6%)	1.090 (0.483-2.459)	p=0.836
CT+TT	351 (29.4%)	120 (31.8%)	1.275 (0.958-1.695)	p=0.096

The adjusted odds ratio (AOR) with their 95% confidence intervals were estimated by multiple logistic regression models after controlling for age, betel quid chewing, cigarette smoking, and alcohol drinking.

**Table 3:** Clinical statuses and genotypic frequencies of *FOSL1* rs637571, rs653914 and rs10896065 in 377 male oral tongue squamous cell carcinoma patients.

rs637571 (N=377)	CC (N=214)	CT + TT (N=163)	AOR (95% CI)	p value
Clinical Stage				
Stage I+II	94 (43.9%)	73 (44.8%)	1.000 (reference)	0.887
Stage III+IV	120 (56.1%)	90 (55.2%)	0.971 (0.644-1.464)	
Tumor size				
≤ T2	105 (49.1%)	88 (54.0%)	1.000 (reference)	0.346
> T2	109 (50.9%)	75 (46.0%)	0.821 (0.545-1.237)	
Lymph node metastasis				
No	131 (61.2%)	99 (60.7%)	1.000 (reference)	0.860
Yes	83 (38.8%)	64 (39.3%)	1.038 (0.682-1.582)	
Cell differentiation				
Well	26 (12.1%)	16 (9.8%)	1.000 (reference)	0.485
Moderate or poor	188 (87.9%)	147 (90.2%)	1.266 (0.653-2.455)	
rs653914 (N=377)	AA (N=104)	AG + GG (N=273)	AOR (95% CI)	p value
Clinical Stage				
Stage I+II	46 (44.2%)	121 (44.3%)	1.000 (reference)	0.977
Stage III+IV	58 (55.8%)	152 (55.7%)	1.007 (0.638-1.589)	
Tumor size				
≦ T2	54 (51.9%)	139 (50.9%)	1.000 (reference)	0.836
> T2	50 (48.1%)	134 (49.1%)	1.049 (0.666-1.653)	
Lymph node metastasis				
No	66 (63.5%)	164 (60.1%)	1.000 (reference)	0.460
Yes	38 (36.5%)	109 (39.9%)	1.194 (0.746-1.914)	
Cell differentiation				
Well	13 (12.5%)	29 (10.6%)	1.000 (reference)	0.627
Moderate or poor	91 (87.5%)	244 (89.4%)	1.190 (0.590-2.400)	
rs10896065 (N=377)	CC (N=257)	CT + TT (N=120)	AOR (95% CI)	p value
Clinical Stage				
Stage I+II	124 (48.2%)	43 (35.8%)	1.000 (reference)	0.027*
Stage III+IV	133 (51.8%)	77 (64.2%)	1.663 (1.059-2.611)	
Tumor size				
≦ T2	143 (55.6%)	50 (41.7%)	1.000 (reference)	0.009*
> T2	114 (44.4%)	70 (58.3%)	1.813 (1.162-2.831)	
Lymph node metastasis				
No	161 (62.6%)	69 (57.5%)	1.000 (reference)	0.385
Yes	96 (37.4%)	51 (42.5%)	1.220 (0.779-1.912)	
Cell differentiation				
Well	31 (12.1%)	11 (9.2%)	1.000 (reference)	0.437
Moderate or poor	226(87.9%)	109 (90.8%)	1.337 (0.643-2.783)	

The adjusted odds ratio (AOR) with their 95% confidence intervals were estimated by multiple logistic regression models after controlling for age, betel quid chewing, cigarette smoking, and alcohol drinking. * p value < 0.05 as statistically significant.

**Table 4:** Clinical statuses and genotypic frequencies of *FOSL1* rs10896065 in 377 oral tongue squamous cell carcinoma patients who are betel quid chewers or not betel quid chewers.

	Non-Betel Quid Chewers (N=128)				Betel Quid Chewers (N=249)			
Variable	CC (N=78)	CT + TT (N=50)	AOR (95% CI)	p value	CC (N=179)	CT + TT (N=70)	AOR (95% CI)	p value
**Clinical Stage**								
Stage I+II	35 (44.9%)	20 (40.0%)	1.000 (reference)	0.618	89 (49.7%)	23 (32.9%)	1.000 (reference)	0.017*
Stage III+IV	43 (55.1%)	30 (60.0%)	1.216 (0.563-2.625)		90 (50.3%)	47 (67.1%)	**2.030 (1.133-3.637)**	
**Tumor size**								
≤ T2	42 (53.8%)	23 (46.0%)	1.000 (reference)	0.262	101 (56.4%)	27 (38.6%)	1.000 (reference)	0.011*
> T2	36 (46.2%)	27 (54.0%)	1.551 (0.720-3.341)		78 (43.6%)	43 (61.4%)	**2.107 (1.187-3.740)**	
**Lymph node metastasis**								
No	44 (56.4%)	28 (56.0%)	1.000 (reference)	0.982	117 (65.4%)	41 (58.6%)	1.000 (reference)	0.409
Yes	34 (43.6%)	22 (44.0%)	0.992 (0.466-2.108)		62 (34.6%)	29 (41.4%)	1.275 (0.717-2.266)	
**Cell differentiation**								
Well	6 (7.7%)	8 (16.0%)	1.000 (reference)	0.085	25 (14.0%)	3 (4.3%)	1.000 (reference)	0.044*
Moderate or poor	72 (92.3%)	42 (84.0%)	0.352 (0.108-1.155)		154(86.0%)	67 (95.7%)	**3.549 (1.032-12.201)**	

The adjusted odds ratio (AOR) with their 95% confidence intervals were estimated by multiple logistic regression models after controlling for age, cigarette smoking, and alcohol drinking. * p value < 0.05 as statistically significant.

**Table 5:** Clinical statuses and gentypic frequencies of *FOSL1* rs10896065 in 377 oral tongue squamous cell carcinoma patients who are smokers and non-smokers.

	Non-smoker (N=88)				smoker (N=289)			
Variable	CC (N=61)	CT + TT (N=27)	AOR (95% CI)	p value	CC (N=196)	CT + TT (N=93)	AOR (95% CI)	p value
**Clinical Stage**								
Stage I+II	28 (45.9%)	11 (40.7%)	1.000 (reference)	0.731	96 (49.0%)	32 (34.4%)	1.000 (reference)	0.028*
Stage III+IV	33 (54.1%)	16 (59.3%)	1.177 (0.465-2.982)		100 (51.0%)	61 (65.6%)	**1.800 (1.066-3.042)**	
**Tumor size**								
≤ T2	33 (54.1%)	10 (37.0%)	1.000 (reference)	0.149	110 (56.1%)	40 (43.0%)	1.000 (reference)	0.033*
> T2	28 (45.9%)	17 (63.0%)	2.004 (0.780-5.151)		86 (43.9%)	53 (57.0%)	**1.751 (1.048-2.926)**	
**Lymph node metastasis**								
No	34 (55.7%)	15 (55.6%)	1.000 (reference)	0.885	127 (64.8%)	54 (58.1%)	1.000 (reference)	0.440
Yes	27 (44.3%)	12 (44.4%)	0.933 (0.365-2.387)		69 (35.2%)	39 (41.9%)	1.229 (0.729-2.072)	
**Cell differentiation**								
Well	6 (9.8%)	3 (11.1%)	1.000 (reference)	0.769	25 (12.8%)	8 (8.6%)	1.000 (reference)	0.372
Moderate or poor	55 (90.2%)	24 (88.9%)	0.798 (0.178-3.579)		171(87.2%)	85 (91.4%)	1.480 (0.626-3.498)	

The adjusted odds ratio (AOR) with their 95% confidence intervals were estimated by multiple logistic regression models after controlling for age, betel quid chewing, and alcohol drinking. * p value < 0.05 as statistically significant.
